# Phylogenetic Groups of *Escherichia coli* Strains from Patients with Urinary Tract Infection in Iran Based on the New Clermont Phylotyping Method

**DOI:** 10.1155/2015/846219

**Published:** 2015-01-08

**Authors:** Darioush Iranpour, Mojtaba Hassanpour, Hossein Ansari, Saeed Tajbakhsh, Gholamreza Khamisipour, Akram Najafi

**Affiliations:** ^1^Department of Internal Medicine, Bushehr University of Medical Sciences, Bushehr 7514633196, Iran; ^2^Department of Genetics, Bushehr University of Medical Sciences, Bushehr 7514633196, Iran; ^3^Department of Genetics, Islamic Azad University, Marvdasht Branch, Marvdasht, Iran; ^4^Department of Microbiology and Parasitology, Faculty of Medicine, Bushehr University of Medical Sciences, Bushehr 7514633196, Iran; ^5^Department of Hematology, Bushehr University of Medical Sciences, Bushehr 7514633196, Iran; ^6^Department of Marine Microbiology, The Persian Gulf Marine Biotechnology Medicine Research Center, Bushehr University of Medical Sciences, Bushehr 7514633196, Iran

## Abstract

*Objectives.* In 2013, Clermont classified *E. coli* strains into eight phylogenetic groups using a new quadruplex PCR method. The aims of this study were to identify the phylogenetic groups of *E. coli* based on this method and to assess their antibiotic resistance patterns in Bushehr, Iran. *Methods*. In this cross-sectional study, 140 *E. coli* isolates were subjected to phylogenetic typing by a quadruplex PCR method. Antimicrobial susceptibility testing was performed by disk diffusion method. *Results*. Phylogenetic group B2 was most predominant (39.3%), followed by unknown (27.1%), E (9.3%), C and clade I (each 6.4%), B1 (5%), F and D (each 2.9%), and A (0.7%). The most common antibiotic resistance was related to amoxicillin (82.1%) and the least to meropenem (0.7%). 82.14% of isolates were multiple drug resistant (MDR). Antibiotic resistance was mainly detected in group B2 (50%). *Conclusions.* Our findings showed the high prevalence of MDR *E. coli* isolates with dominance of group B2. About 25% of *E. coli* isolates belong to the newly described phylogroups C, E, F, and clade I. Such studies need to be done also in other regions to provide greater understanding of the antibiotic resistance pattern and the prevalences of different phylogenetic groups.

## 1. Introduction

Urinary tract infections (UTIs) are among the most frequent bacterial infectious diseases, affecting both inpatients and outpatients around the world [1].* Escherichia coli* is one of the most predominant pathogens, causing 80–90% of all episodes of UTIs [1–3] and is a troublesome health problem in many different countries worldwide [4]. The frequency of UTI is affected by sex and age, with UTI most commonly found in females of all age groups [2].

Clermont and colleagues developed a triplex PCR assay to detect the genes* chuA*,* yjaA*, and TspE4.C2 in 2000 [5]. Regarding the presence/absence of these three genes, an* E. coli* strain could be classified into one of the main phylogroups, A, B1, B2, or D [5]. The growing body of multilocus sequence type (MLST) data for* E. coli* reported from different hosts and habitats demonstrated that 80 to 85% of the phylogroup classifications are correct. However, a fraction of strains with special triplex PCR genotypes (A0, D1, and D2) were incorrectly assigned [6]. In 2013 Clermont and colleagues added an additional gene target,* arpA*, to those three candidate markers and made a quadruplex PCR to classify an* E. coli* isolate into one of the phylogroups A, B1, B2, C, D, E, F, and clade I [6].

Worldwide phylogenetic analyses have demonstrated that virulent extraintestinal* E. coli* strains belong mainly to group B2 and, to a lesser extent, to group D. In contrast, most of the commensal strains are associated with group A or group B1 [1, 2, 7, 8].

Sulfamethoxazole-trimethoprim (cotrimoxazole), fluoroquinolones, beta-lactams, nitrofurantoin, and fosfomycin are the most important antibiotics used in the therapy of UTI in both community and hospital settings [1, 2]. Currently, studies clearly show that there is an increasing resistance to conventional antibiotics among* E. coli* strains causing UTIs [9, 10]. Additionally, in recent years the difference in the antibacterial resistance of* E. coli* strains of particular phylogenetic groups has been considered a matter of importance [11].

Antimicrobial susceptibility surveillance is essential to evaluate the size of the problem and for the appropriate selection of antimicrobial drugs for treating infected patients [9, 10].

To our knowledge this is the first study utilizing the new quadruplex PCR method to categorize phylogenetic groups of* E. coli* isolated from patients with UTI in Bushehr, Iran. The aims of this study were to identify the phylogenetic groups of* E. coli* based on new Clermont's method and to assess the antimicrobial resistance profile of these strains.

## 2. Materials and Methods

### 2.1. Sample Collection

In this cross-sectional study a total of 6406 urine specimens were collected from patients with or without clinical symptoms related to urinary tract infection (UTI), admitted to the Shohadaye Khalije Fars and 17 Shahrivar Hospitals and 5 private clinics (Hakim, Mehr, Central laboratory, Navid, and Pasteur) in Bushehr, Iran, in 2013. The European Urinalysis Guidelines have explained the limit for symptomatic UTI caused by* E. coli* to be 10^5^ CFU/mL [12]. All the samples were transported to the Microbiology Laboratory at Bushehr University of Medical Science for further evaluation under sterile conditions. Also, demographic data such as age and sex, history of urinary infection, and history of antibiotic usage were collected. All steps of this study were approved by the Ethics Committee of Bushehr University of Medical Science.

### 2.2. Bacterial Isolates

140 unique* E. coli* isolates (obtained in monoinfection) collected over a period of one year were selected for this study. All these isolates were cultured on standard media, including blood agar, MacConkey‘s agar, and Eosin Methylene Blue (Merck, Germany), and were incubated at 37°C for 24 hours. The isolation and identification of* E. coli* strains were performed by routine biochemical tests, that is, indole, motility, methyl red, Voges-Proskauer, lysine decarboxylase, Simmon citrate agar, and triple sugar iron agar (TSI) [13]. In addition, other Gram negative bacteria were identified by standard biochemical methods [13]. In this study, a sample of each stored strain was evaluated for purity on MacConkey agar and kept at −20°C in skim milk (Merck, Germany) for long-term storage.

### 2.3. Antimicrobial Susceptibility Testing

Susceptibility testing was performed by the Kirby-Bauer disk diffusion method on Mueller Hinton agar (Merck, Germany) according to the definition of the Clinical Laboratory Standard Institute [14]. The antimicrobial drugs used were ampicillin (10 *μ*g), amikacin (30 *μ*g), amoxicillin (25 *μ*g), ceftizoxime (30 *μ*g), ceftazidime (30 *μ*g), cephalothin (30 *μ*g), ciprofloxacin (5 *μ*g), cefotaxime (30 *μ*g), ceftriaxone (30 *μ*g), gentamicin (10 *μ*g), imipenem (10 *μ*g), levofloxacin (5 *μ*g), meropenem (10 *μ*g), norfloxacin (10 *μ*g), nalidixic acid (30 *μ*g), nitrofurantoin (300 *μ*g), cotrimoxazole (sulfamethoxazole 23.75 *μ*g + trimethoprim 1.75 *μ*g), and ofloxacin (5 *μ*g), (Padtan Teb, Tehran, Iran).* E. coli* ATCC 25922 and ATCC 35218 were used as the quality control strains.

### 2.4. Isolation of Bacterial DNA

DNA for amplification was extracted from whole cells by the boiling method as follows. A full loop of pure colonies and overnight cultures was suspended in 1 mL of sterile distilled water. The cells were lysed by heating at 95°C for 10 minutes. The supernatant was harvested by centrifugation at 12,000 rpm for 5 minutes and kept at −20°C. The supernatant was used in subsequent PCR as template DNA [15].

The integrity of extracted DNA was evaluated by electrophoresis on 1% agarose gel. Also, the purity of DNA was determined by the ratio A260/A280 in a Biophotometer (Eppendorf, Germany).

### 2.5. Phylogenetic Analysis

The distribution of phylogenetic groups amongst* E. coli* isolates was determined as recently described by Clermont and colleagues [6]. Briefly, a single reaction mixture contained 2 *μ*L of 10x buffer (supplied with* Taq* polymerase), 2 *μ*L of DNA (approximately 100 ng), 20 pmol of each appropriate primer (except for AceK.f (40 pmol), ArpA1.r (40 pmol), trpBA.f (12 pmol), and trpBA.r (12 pmol)) (Shanghai Generay Biotech Co., Ltd.), 2 mM of each dNTP, and 2 U of Taq DNA polymerase (Fermentas, Lithuania) in a total volume of 20 *μ*L. Primer sequences for the new phylogroup assignment method are shown in Table 1.

PCR amplifications were carried out on a thermal cycler Mastercycler gradient (Eppendorf, USA) under the following conditions: initial denaturation at 94°C for 4 min and 30 cycles for each denaturation at 94°C for 5 sec, annealing at 57°C for 20 sec (group E) or 59°C for 20 sec (quadruplex and group C), amplification at 72°C for 1 min, and final extension at 72°C for 5 min [6]. PCR products were analyzed by electrophoresis with a 2% agarose gel, stained with DNA safe stain (CinnaGen, Tehran, Iran) and visualized using Gel Doc 2000 transilluminator (Bio-Rad Laboratories, Milan, Italy). A molecular weight standard (50 bp ladder, Fermentas, Lithuania) was included on each gel.

### 2.6. Statistical Analysis

The data was statistically analyzed using SPSS version 18.0 (SPSS Inc., Chicago, IL, USA). The chi-square test or the Fisher exact test was applied to compare categorical variables. *P* values < 0.05 were considered to be statistically significant.

## 3. Results

A total of 356 positive urine specimens* E. coli* strains were isolated in 140 cases (39.33%) with a count of 10^5^ CFU/mL. In this study the contribution of other Gram negative bacteria was* Klebsiella* (25.29%),* Proteus* (12.36%),* Pseudomonas* (8.99%),* Enterobacter* (5.62%),* Citrobacter* (3.65%),* Acinetobacter* (2.81%), and* Moraxella* (1.97%), respectively.* E. coli* strains were detected more often in females (112/140; 80%) than in males (28/140; 20%) (*P* = 0.001). The participation of hospital- and community-acquired strains of* E. coli* was 55.7% (78 cases) and 44.3% (62 cases), respectively. All the patients with UTI ranged between the ages of 1 and 91 years and were divided into four specific age groups (e.g., less than 15, 15 to 45, 45 to 60 and more than 60 years). The median age was 34.39 ± 22.72 years. The age group analysis revealed that patients younger than 45 years accounted for 70% of the overall UTI disease, with those between 15 and 45 years of age being the most affected (50%) (*P* = 0.001).

**Figure 1:**
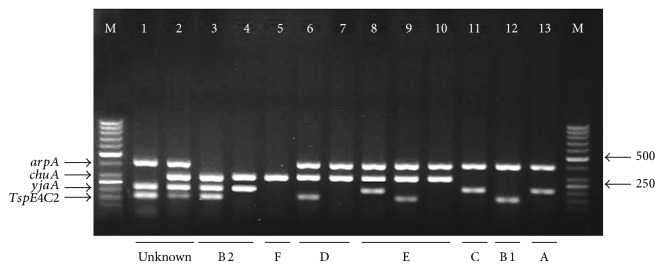
Quadruplex PCR profiles of new Clermont phylotyping method. Lane 1, unknown (+ − + +); lane 2, unknown (+ + + +); lane 3, group B2 (− + + +); lane 4, group B2 (− + + −); lane 5, group F (− + −  −); lane 6, group D (+ + − +); lane 7, group D (+ + −  −); lane 8, group E (+ + + −); lane 9, group E (+ + − +); lane 10, group E (+ + −  −); lane 11, group C (+ − + −); lane 12, group B1 (+ −  − +); lane 13, group A (+ − + −). M: molecular weight marker (50 bp, Fermentas).

In the present study, the predominant phylogenetic group was B2 (55/140; 39.3%), followed by unknown (38/140; 27.1%), E (13/140; 9.3%), C and clade I (9/140; each 6.4%), B1 (7/140; 5%), F and D (4/140; each 2.9%), and A (1/140; 0.7%) (*P* = 0.001) (Figure 1). There was no significant difference in the phylogenetic group composition with regard to age or gender of the patients.

The disk diffusion method indicated that the* E. coli* strains had the highest resistance to amoxicillin (82.1%) and ampicillin (80%) while resistance to meropenem (0.7%) and nitrofurantoin (1.4%) was minimal (Table 2).

Regarding the antibiotic classes it was recognized that most antibiotic resistance was related to sulfonamides (57.9%), followed by *β*-lactams (48.67%), quinolones (36.72%), carbapenems (15%), aminoglycosides (12.8%), and nitrofurans (1.4%), respectively.

In this study 82.14% (115 cases) of isolates were considered multiple drug resistant (MDR) and 45.71% (64 cases) of the investigated strains were resistant to at least seven of the examined antibiotics. Only 12.14% of the strains were susceptible to all studied antibiotics.

Of these, phylotyping analysis revealed that the most prevalent multiple drug resistant strain belonged to phylogroup B2 (50%) (*P* = 0.001). We found significant differences between phylogenetic groups and resistance to all the studied antibiotics except nitrofurantoin, amikacin, and meropenem.

Group B2 isolates were totally resistant to all studied antibiotics except meropenem. Group D isolates were only resistant to four drugs, namely, gentamycin, ampicillin, amoxicillin, and trimethoprim-sulfamethoxazole (Table 2).

## 4. Discussion

Currently, the clinical management of UTI is a major global problem because of the increase in infections caused by* E. coli* strains that have acquired resistance to commonly used antimicrobial agents [16]. Studies have indicated that* E. coli* antibiotic resistance is mainly related to the phylogenetic grouping [17]. The aim of this study was to identify the phylogenetic groups of* E. coli* based on the new Clermont method and to assess the relationship between these phylogroups and antibiotic resistance patterns.

The previous triplex PCR method differentiates all* E. coli* strains into four phylogroups, A, B1, B2, and D [5]. Extended phylogenetic analyses have shown that virulent extraintestinal* E. coli* strains belonged typically to group B2 and less often to group D [1, 7, 8]. Our findings are in accordance with other studies in Iran [18] and worldwide [11, 19, 20] where it was found that the majority of isolates of* E. coli* predominantly belong to phylogenetic group B2.

In this study, group A had the lowest prevalence. This result is in contrast to those noted in some previous studies of extraintestinal* E. coli* which indicated that most of isolates belonged to group A [15, 21, 22].

The prevalences of the D and B1 phylogroups found in our study are much lower than in other countries [3, 19, 22].

These different prevalences of the phylogenetic groups reported in different studies may be explained by the health status of the host, dietary and host genetic factors, environmental, social, and geographic conditions, or differences in sampling areas [15].

The most significant advantage of the new Clermont quadruplex PCR method for* E. coli* phylogroup assignment is its ability to classify strains into groups C, E, F, and clade I. In the present study, about 25% of* E. coli* isolates belonged to these phylogroups. Therefore, this is the first study utilizing a new quadruplex PCR method for phylotyping of* E. coli* in UTI isolates. No data about the prevalences of the newly described phylogroups in different countries around the world is available.

Clermont et al. demonstrated that only 1% of* E. coli* strains could not be assigned to one of the eight recognized phylogroups using the extended quadruplex method [6]. However, in the present study more than a quarter (27.1%) of* E. coli* isolates of patients with UTI remained unclassified. This finding is difficult to explain but probably these unassignable strains are extremely rare phylogroups or are the result of a recombination between two different phylogroups [6].

In this study, 80% of* E. coli* strains were resistant to ampicillin. The high frequency of ampicillin resistance among* E. coli* isolates has also been recently reported in various Asian and European countries, including Iran [23], China [24], Switzerland [25], and Italy [26], indicating that treatment with these agents may be inadequate in many cases.

Recently, trimethoprim-sulfamethoxazole has been considered as an effective drug in the treatment of UTIs in many developed and developing countries [27–29]. However, our results show relatively high resistance rates (57.9%) to this antibiotic. This high resistance may be attributed to the possibility of widespread use of this low cost antimicrobial drug for treatment of UTI in the region.

In the present study, there were high susceptibility rates to meropenem and nitrofurantoin, indicating that carbapenem and nitrofuran resistance is still an unusual phenotype among* E. coli* isolates. These antibiotics may be considered as good alternative agents for the treatment of UTI in the region. This finding was similar to those demonstrated in other surveillance studies, in which these antibiotics were the most effective antimicrobial drugs for the treatment of UTI [26, 30, 31]. Resistance to commonly used antimicrobial drugs raises the global concern about the increased clinical failure rates and the limited therapeutic options available for physicians treating UTIs [32].

Our study showed that most group B2 isolates were resistant to all studied antibiotics except meropenem. This finding is in agreement with other studies in Iran [33] and Spain [27] where it was reported that phylogenetic group B2 is the most drug resistant.

In addition, all phylogenetic group D isolates were resistant to quinolones. Also, group D isolates were only resistant to four drugs. In comparison with our results, Bashir et al. reported that group D was dominant with regard to drug resistance [34].

In conclusion, our findings showed that group B2 was the most predominant phylogenetic group and most resistant strain to commonly used antibiotics among patients with UTI. About 25% of* E. coli* isolates belonged to the newly described phylogroups C, E, F, and clade I. Studies like ours need to be done also in other regions to provide greater understanding of the prevalences and geographic distribution of* E. coli* phylogenetic groups.

Regular monitoring of antibiotic resistance patterns will also help clinicians to prescribe the most appropriate antibiotic and to avoid further development of antimicrobial drug resistance.

## Figures and Tables

**Table 1:** Primer sequences used in the extended quadruplex phylotyping method [6].

PCR reaction	Primer ID	Target	Primer sequence	PCR product (bp)
Quadruplex	chuA.1b	*chuA *	5-ATGGTACCGGACGAACCAAC-3	288
	chuA.2		5-TGCCGCCAGTACCAAAGACA-3	
	yjaA.1b	*yjaA *	5-CAAACGTGAAGTGTCAGGAG-3	211
	yjaA.2b		5-AATGCGTTCCTCAACCTGTG-3	
	TspE4C2.1b	*TspE4C2 *	5-CACTATTCGTAAGGTCATCC-3	152
	TspE4C2.2b		5-AGTTTATCGCTGCGGGTCGC-3	
	AceK.f	*arpA *	5-AACGCTATTCGCCAGCTTGC-3	400
	ArpA1.r		5-TCTCCCCATACCGTACGCTA-3	

Group E	ArpAgpE.f	*arpA *	5-GATTCCATCTTGTCAAAATATGCC-3	301
	ArpAgpE.r		5-GAAAAGAAAAAGAATTCCCAAGAG-3	

Group C	trpAgpC.1	*trpA *	5-AGTTTTATGCCCAGTGCGAG-3	219
	trpAgpC.2		5-TCTGCGCCGGTCACGCCC-3	

Internal control	trpBA.f	*trpA *	5-CGGCGATAAAGACATCTTCAC-3	489
	trpBA.r		5-GCAACGCGGCCTGGCGGAAG-3	

**Table 2:** Prevalence of resistance among various phylogenetic groups of *E. coli* isolates.

Antibiotics by class	Antibiotic	Phylogenetic group									Total
		B1	B2	F	U	D	E	Clade I	C	A	
Aminoglycosides	Gentamycin	—	15 (10.7)	3 (2.1)	5 (3.6)	1 (0.7)	3 (2.1)	1 (0.7)	1 (0.7)	—	29 (20.7)
	Amikacin	—	5 (3.6)	—	1 (0.7)	1 (0.7)	—	—	—	—	7 (5)

Beta-lactams	Amoxicillin	4 (2.9)	50 (35.7)	3 (2.1)	31 (22.1)	2 (1.4)	9 (6.4)	9 (6.4)	6 (4.3)	1 (0.7)	115 (82.1)
	Ampicillin	4 (2.9)	49 (35)	3 (2.1)	30 (21.4)	1 (0.7)	9 (6.4)	9 (6.4)	6 (4.3)	1 (0.7)	112 (80)
	Cephalothin	—	32 (22.9)	3 (2.1)	15 (10.7)	—	5 (3.6)	3 (2.1)	4 (2.9)	1 (0.7)	63 (45)
	Ceftriaxone	—	29 (20.7)	3 (2.1)	15 (10.7)	—	5 (3.6)	3 (2.1)	4 (2.9)	1 (0.7)	60 (42.9)
	Cefotaxime	—	29 (20.7)	3 (2.1)	14 (10)	—	5 (3.6)	3 (2.1)	3 (2.1)	1 (0.7)	58 (41.4)
	Ceftazidime	—	22 (15.7)	2 (1.4)	10 (7.1)	—	4 (2.9)	3 (2.1)	2 (1.4)	1 (0.7)	44 (31.4)
	Ceftizoxime	—	14 (10)	1 (0.7)	3 (2.1)	—	2 (1.4)	3 (2.1)	1 (0.7)	1 (0.7)	25 (17.9)

Sulfonamides	Trimethoprim-sulfamethoxazole	1 (0.7)	32 (22.9)	2 (1.4)	25 (17.9)	2 (1.4)	7 (5)	6 (4.3)	5 (3.6)	1 (0.7)	81 (57.9)

Quinolones	Nalidixic acid	1 (0.7)	30 (21.4)	3 (2.1)	16 (11.4)	—	5 (3.6)	5 (3.6)	2 (1.4)	1 (0.7)	63 (45)
	Levofloxacin	1 (0.7)	26 (18.6)	3 (2.1)	11 (7.9)	—	5 (3.6)	1 (0.7)	2 (1.4)	1 (0.7)	50 (35.7)
	Norfloxacin	1 (0.7)	26 (18.6)	3 (2.1)	10 (7.1)	—	5 (3.6)	—	2 (1.4)	1 (0.7)	48 (34.3)
	Ofloxacin	1 (0.7)	25 (17.9)	3 (2.1)	10 (7.1)	—	5 (3.6)	1 (0.7)	2 (1.4)	1 (0.7)	48 (34.3)
	Ciprofloxacin	1 (0.7)	26 (18.6)	3 (2.1)	9 (6.4)	—	5 (3.6)	1 (0.7)	2 (1.4)	1 (0.7)	48 (34.3)

Carbapenem	Imipenem	—	23 (16.4)	3 (2.1)	7 (5)	—	4 (2.9)	1 (0.7)	2 (1.4)	1 (0.7)	41 (29.3)
	Meropenem	—	—	—	—	—	—	1 (0.7)	—	—	1 (0.7)

Nitrofurans	Nitrofurantoin	—	1 (0.7)	—	—	—	—	1 (0.7)	—	—	2 (1.4)

^*^Numbers in parenthesis are percentages.
